# Cyanoacrylate Adhesive in Free Gingival Graft Healing: A Digital and Microcirculatory Randomized Clinical Trial

**DOI:** 10.1111/jerd.13516

**Published:** 2025-07-07

**Authors:** Zeynep Turgut Çankaya, Sühan Gürbüz, Evşen Tamam

**Affiliations:** ^1^ Department of Periodontology Faculty of Dentistry, University of Gazi Ankara Turkey; ^2^ Department of Prosthodontics Faculty of Dentistry, University of Gazi Ankara Turkey

**Keywords:** cyanoacrylate, free gingival graft, graft shrinkage, laser doppler flowmetry, periodontal surgery, tissue adhesives, wound healing

## Abstract

**Introduction:**

This study aimed to evaluate the efficacy of n‐butyl cyanoacrylate (CA) in stabilizing free gingival grafts (FGGs) without sutures at the recipient site and accelerating postoperative healing at the donor site. Additionally, perfusion dynamics and graft shrinkage were assessed using laser Doppler flowmetry (LDF) and digital impression techniques.

**Methods:**

This randomized controlled clinical trial included 40 systemically healthy, non‐smoking patients with insufficient keratinized tissue in the mandibular region. Patients were randomly assigned to the test group (FGG stabilized with CA) or the control group (FGG secured with sutures). Periodontal parameters, intraoperative durations, LDF values at recipient and donor sites, graft shrinkage (digital impression), and postoperative pain (VAS scores) were recorded.

**Results:**

Graft shrinkage was significantly lower in the CA group (37.23%) than in the control group (45.15%) (*p* < 0.001). The test group exhibited significantly higher LDF values at the donor site on days 4 and 7, suggesting enhanced perfusion and early vascularization (*p* = 0.002, *p* < 0.001). Postoperative pain (VAS scores) was significantly lower in the CA group until day 6 (*p* < 0.05). Furthermore, FGG stabilization time was three times shorter in the CA group than in the control group (*p* < 0.001). By day 30, perfusion values equalized between groups, indicating that CA did not negatively impact long‐term tissue healing.

**Conclusion:**

N‐butyl cyanoacrylate provides a rapid, reliable, and minimally traumatic alternative to sutures for FGG stabilization. Its use may reduce graft shrinkage, enhance early vascularization, minimize postoperative pain, and shorten surgical time. These findings support CA as a promising wound dressing and stabilizer in periodontal plastic surgery. However, further randomized clinical trials with larger sample sizes are needed to evaluate its long‐term effects.

## 1 Introduction

Free gingival graft (FGG) is widely used in periodontal plastic surgery to treat gingival recession, increase keratinized tissue width, and enhance peri‐implant soft tissue health. By forming a resilient keratinized barrier, FGG improves oral hygiene, resists frictional forces, and stabilizes mucosal tissue. Due to its predictability and broad applicability, it remains the gold standard in periodontal plastic surgery [1].

FGG involves two wound sites: the recipient and donor areas. The donor site, typically the hard palate, heals by secondary intention and is a key focus due to postoperative pain, bleeding, and discomfort. Recent studies explore biomaterials, growth factors, and biologics such as platelet‐rich fibrin to enhance healing and reduce complications [2]. Salivary cytokines, growth factors, and proteases facilitate oral wound healing, emphasizing the need for optimized strategies.

Covering the donor site is essential to prevent contamination, promote healing, and reduce pain. Strategies include biopolymers (hyaluronic acid, collagen matrix, chitosan) [3, 4, 5], growth factors (platelet‐rich fibrin, platelet rich plasma) [6, 7, 8], plant‐derived agents (medicinal herbs, ozonated olive oil, *Moringa oleifera* ) [9, 10, 11], chemotherapeutic agents (chlorhexidine gluconate, sodium hypochlorite, povidone‐iodine, hydrogen peroxide) [12], laser therapies (diode, low‐level laser) [13, 14], and electrical stimulation [15].

Graft adaptation and long‐term stability are critical for FGG success. Recent studies highlight the role of recipient site biology, vascularization, and integration [16]. Growth factors, biomimetic materials, and surgical modifications improve stability and reduce complications.

FGGs are traditionally stabilized using sutures, but needle‐induced trauma contributes to graft contraction. Hematoma formation due to excessive suturing exacerbates contraction. Alternative suture‐free techniques, including fibrin glue, staples, adhesive tapes, and cyanoacrylate‐based adhesives, have gained interest [17, 18, 19]. Cyanoacrylate‐based adhesives s offer strong adhesion, moisture resistance, and bacteriostatic properties, first proposed for FGG stabilization in 1978 [20].

Cyanoacrylate‐based adhesives polymerize upon contact with water, mucosa [21], skin, blood, or bone, promoting rapid hemostasis [22] and potentially reducing postoperative bleeding [23] at the donor site. However, previous studies assumed uniform FGG shrinkage, which may be inaccurate as graft edges do not always remain rectangular [24, 25, 26, 27]. Digital impression techniques allow for more precise evaluation of shrinkage by considering morphological changes.

Early vascularization is crucial for FGG survival. The first 48 h rely on plasmic circulation, with anastomosis forming by day 3 and vascular layers developing by day 5, leading to sinusoidal vascular loops [28, 29].

Laser Doppler flowmetry (LDF) is a non‐invasive tool for assessing blood flow and vascular changes in studies investigating materials influencing early‐stage FGG microcirculation [30]. LDF provides objective, repeatable, and quantitative data on gingivitis [31], periodontitis [32], and periodontal wound healing [33]. Recent studies have expanded its application to peri‐implant mucosal assessment [34] and palatal donor site wound healing following mucogingival procedures [35].

Studies investigating shrinkage in free gingival grafts (FGGs) have primarily focused on evaluating the dimensional reduction observed after the procedure. For example, the study by Gümüş and Buduneli [25] was one of the earlier efforts to explore this phenomenon. Over time, the number of studies examining the influence of different graft stabilization techniques on shrinkage has increased. However, assessing graft shrinkage merely as a numerical or surface‐level outcome is not sufficient; understanding the underlying biological mechanisms that contribute to this process is equally essential. Accordingly, recent studies have started to investigate the vascular and physiological aspects of graft integration, highlighting the importance of biologically informed evaluation methods. Regarding the donor site, previous studies have predominantly evaluated the effects of cyanp.

The rationale for the present study arises from this need. Through the use of LDF, this study not only quantifies the amount of graft contraction but also investigates the physiological basis for the observed differences, particularly the role of early microcirculatory dynamics in graft integration. This approach aims to move beyond outcome‐based evaluation by providing a mechanistic perspective on graft stabilization, ultimately contributing to more predictable and evidence‐based clinical decision‐making.

This study evaluates the efficacy of cyanoacrylate in securing an FGG without sutures at the recipient site and its effect on postoperative healing at the donor site. Healing was assessed using LDF and digital impression techniques to analyze blood flow and tissue response. Although previous studies investigated dimensional and vascular changes in FGG healing, this is the first study to evaluate absolute area changes in cyanoacrylate‐secured FGGs using digital impression techniques and its impact on microcirculation at both donor and recipient sites.

This randomized controlled clinical trial compares sutures vs. cyanoacrylate adhesives for FGG stabilization during early healing. Graft shrinkage, microcirculation, and postoperative pain were evaluated. The study also examines cyanoacrylate's role in promoting early vascularization and reducing contraction.

## 2 Materials and Methods

This prospective, parallel‐group, randomized clinical trial followed CONSORT guidelines to assess patient discomfort, pain, and the vascular effects of cyanoacrylate after FGG harvesting.

### 2.1 Study Population

Forty systemically healthy, non‐smoking patients (19 females, 21 males; mean age 32.5 ± 3.4 years) with insufficient keratinized tissue in the mandibular region were recruited at the University of Gazi, Department of Periodontology, Faculty of Dentistry, Ankara, Türkiye. Patients were randomly assigned to either the test group, where the FGG was stabilized with cyanoacrylate, or the control group, where the FGG was secured with sutures.

### 2.2 Inclusion and Exclusion Criteria

Patients were included in the study if they had < 2 mm of keratinized tissue in the mandibular region (central/lateral incisors, canines, premolars), as assessed via a functional test [36]. Additionally, participants were required to have a gingival thickness of ≥ 1 mm, no high frenulum attachment, no parafunctional habits, and no history of mucogingival or periodontal surgery. Patients with restorations or crown prostheses in the recipient area were excluded. To be eligible as a donor site, the palatal tissue thickness had to be at least 3 mm.

Patients were excluded if they had systemic diseases that could affect wound healing, such as uncontrolled diabetes, autoimmune disorders, or osteoporosis. Individuals with active periodontal disease or a history of severe periodontitis were not included. Additionally, smokers or users of smokeless tobacco, as well as those taking anticoagulants, corticosteroids, immunosuppressants, or bisphosphonates, were excluded. Patients who had undergone periodontal surgery within the last 6 months, were pregnant or lactating, or had genetic or connective tissue disorders affecting soft tissue integrity were also not eligible to participate in the study.

This study was approved by the Gazi University Faculty of Dentistry Research and Ethics Committee (No: 84/12.08.2018) and registered at clinicaltrials.gov (NCT6821984), in accordance with the Declaration of Helsinki (1975, revised 2013) [37, 38]. Written informed consent was obtained from all participants after a detailed explanation of the clinical procedures.

### 2.3 Outcome Measures

Periodontal indices, including plaque index (PI) [39], gingival index (GI) [40], probing pocket depth (PPD), and palatal thickness [22] were assessed at six sites per tooth using a Williams periodontal probe. Surgical durations for recipient site preparation, FGG stabilization, and graft harvesting were recorded. LDF was performed at baseline and postoperatively on days 4, 7, 10, 14, and 30, following the methodology described in our previous study [3]. Postoperative pain was evaluated using the visual analog scale (VAS) between days 1 and 7. Graft shrinkage was assessed on day 30 using digital image analysis.

FGG area was measured postoperatively and on day 30. The percentage of FGG area reduction was calculated using the following formula:

$$\begin{array}{c} \mathrm{FGG}\;\text{area reduction}\;\left(\%\right)=\left(\left(\text{Baseline}\;\mathrm{FGG}\;\text{area}\right.\right.\\ \left.-\text{Postoperative}\;\mathrm{FGG}\;\text{area}\;\mathrm{on}\;\mathrm{day}\;30\right)\\ \left./\text{Baseline}\;\mathrm{FGG}\;\text{area}\right)\times 100. \end{array}$$

The primary outcome was the change in FGG area between baseline and day 30. Secondary outcomes included changes in LDF values, VAS scores, and the duration of surgical stages. The sample size was determined based on FGG area data from a previous study [25]. Prior to patient recruitment, a one‐way fixed‐effects analysis of variance with two levels indicated that a sample size of 18 patients would provide 80% power to detect a between‐group effect size of f = 0.489 at a significance level of α = 0.05. To account for potential dropouts, 20 patients were enrolled in each group, all of whom completed the study. The power analysis was conducted using G*Power v.3 software. Patients were randomized in a 1:1 ratio using a computer‐generated randomization scheme, ensuring equal allocation across the two treatment groups. Stratification was performed based on gender and preoperative palatal thickness (≥ 2.5 mm vs. < 2.5 mm) to achieve comparable groups.

### 2.4 Presurgical Treatment

At baseline, full‐mouth (FM) initial periodontal therapy was performed, and oral hygiene instructions were provided. The modified Bass technique and daily interdental brush use were recommended. After 3 weeks, only participants with an FMPI of ≤ 1 and an FMGI of ≤ 1 were included.

### 2.5 Surgical Treatment

FGG procedures were performed using Sullivan's surgical technique, with all grafts placed on a periosteal bed under local anesthesia [28].

### 2.6 Recipient Site Preparation

The rolling technique was used to identify the mucogingival junction by folding the mucosa until attached gingiva was detected with a periodontal probe.

All surgeries were performed under local anesthesia (2% lidocaine with 1:100,000 epinephrine). A No. 15 scalpel was used to make an initial incision along the mucogingival junction, followed by partial‐thickness flap dissection to remove soft tissue and muscle attachments. The recipient site edges were stabilized with 4–0 silk sutures to prevent displacement. The mesiodistal and occlusoapical dimensions of the site were measured to determine the appropriate graft height before placement. To facilitate graft size standardization, all recipient sites were chosen from the mandibular central incisors. All surgical procedures were performed by a calibrated and experienced periodontist with prior training in graft dimension planning and measurement.

### 2.7 Graft Harvesting and Donor Site Preparation

Local anesthesia was administered, and graft dimensions were marked using a periodontal probe. A 1–1.5 mm thick graft was harvested with a No. 15 scalpel, ensuring the palatal artery remained intact. To maintain viability, the graft was immediately immersed in 0.9% saline.

The donor site was rinsed with sterile saline, and hemostasis was achieved using saline‐moistened gauze. In the test group, 0.25 mL of n‐butyl‐2‐cyanoacrylate (TopoCryl Skin Adhesive Bandage, Heal & Care Inc., US) was applied according to the manufacturer's instructions. Activation was performed by pressing the plastic pipette, releasing the adhesive from its internal chamber. Half of the cyanoacrylate was applied as fine microdroplets to ensure full coverage of the donor site. In the control group, the donor site was left to heal by secondary intention after applying saline‐moistened gauze.

Patients were advised to refrain from food and beverages for 1 hour. No wound dressing or acrylic stent was used in either group during healing.

### 2.8 Graft Stabilization

To prevent dead space formation and excessive blood clot accumulation beneath the graft, gentle pressure was applied for 2 min using a saline‐moistened sponge [41]. In the control group, the graft was secured at the coronal mesial and distal corners with 5/0 monofilament sutures. In the test group, cyanoacrylate was applied along the external surface of the FGG edges, forming a uniform 2 mm‐wide adhesive layer specifically at the coronal mesial and distal corners. After confirming graft stability during lip movements, the graft surface was cleaned with a saline‐moistened sponge, and gentle pressure was applied for 1–2 min to ensure adaptation to the recipient site [25]. All grafts were covered with aluminum foil and a periodontal pack (COE‐PAK) for 3 days.

### 2.9 Postsurgical Maintenance

Patients were advised to apply intermittent cold compresses to the surgical area for 24 h and to refrain from brushing in that region. Each patient was prescribed a 0.12% chlorhexidine mouth rinse to be used twice daily for 2 weeks. Following suture removal, patients were instructed to gently brush the surgical area with a soft‐bristled toothbrush for 1 week.

### 2.10 Data Collection

#### 2.10.1 Laser Doppler Flowmetry

To ensure stable blood pressure, measurements were taken after a 5‐min rest in a semi‐inclined dental chair under standardized conditions [42]. Data were collected and analyzed using dedicated software, with results expressed as perfusion units (PU).

Relative blood flow velocity and red blood cell count at the donor and recipient sites were quantified using an LDF device (PeriFlux 5000 Perimed AB) with a flexible fiber‐optic probe (model PF 407, Perimed AB, Sweden) and a probe holder(PH 07–4 tape Fixated Probe Holder). Measurements were performed by an experienced periodontist under technical supervision. Backscattered light was processed to calculate PU, which was displayed in real time on a monitor. A stable perfusion trace (≥ 5 s) was required before recording a reference measurement over a 3‐min period. LDF values were designated as PU‐D (donor site) and PU‐R (recipient site). Each recording lasted approximately 5 s and was analyzed using the PeriSoft software for Windows, version 2.50 (Perimed AB, Sweden), which automatically calculated the mean PU, standard deviation, maximum and minimum values, area under the curve, slope, and percentage change from the first to the last value. To ensure repeatability and minimize motion artifacts, the probe was mounted on a probe holder that allowed for perpendicular and stable contact with the tissue surface at a consistent angle and distance. Measurements were conducted before local anesthesia administration and repeated on postoperative days 4, 7, 10, 14, and 30.

#### 2.10.2 Graft Shrinkage Measurement

**FIGURE 1:**
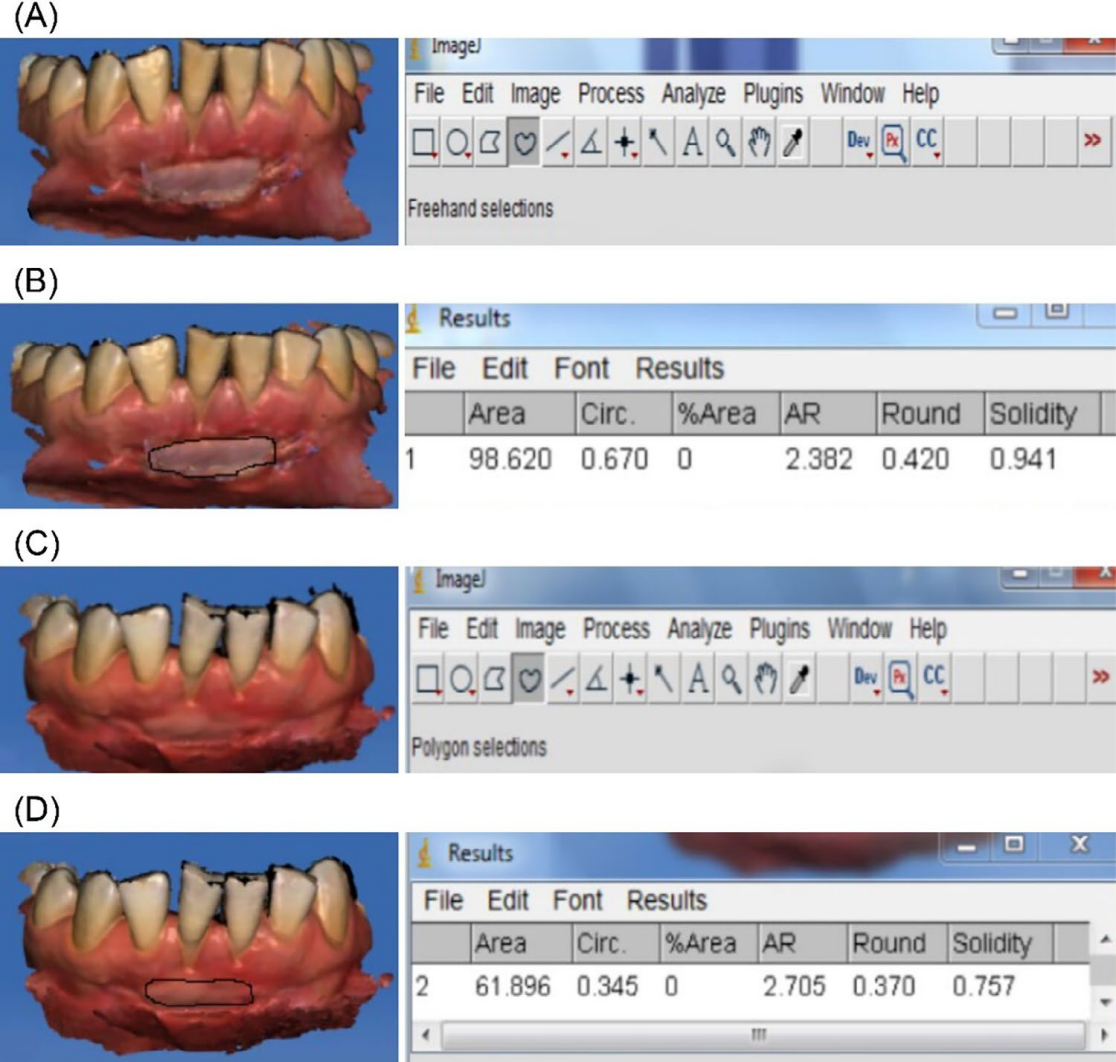
(A) Digital impression obtained on the day of surgery from the CA group. (B) The free gingival graft area was delineated using the Freehand and Polygon tools in ImageJ software. (C) Digital Impression of the FGG Image in the CA Group on Postoperative Day 30. (D) Area Calculation from the Digital Measurement of the FGG Image at Postoperative Day 30.

For each patient, digital impression images were acquired to capture the entire FGG area based on x, y, and z‐axis intersections. Using specialized software (Cerec 4.4, Sirona Dental Systems, Germany), images were aligned to the midline by adjusting grid lines. Standardized frontal view screenshots were taken, generating three images per session and six per patient, totaling 240 images for all 40 patients. These images were analyzed in ImageJ to calculate graft shrinkage percentages. Area measurements were performed by a calibrated periodontist who was blinded to patient group allocation. (Figure 1) Digital impressions were acquired using the CEREC Omnicam intraoral scanner (Dentsply Sirona, Germany) immediately after graft placement and at the 30‐day follow‐up. Standardized screenshots of the 3D models were captured in the CEREC software from a consistent frontal view.

These images were imported into ImageJ (NIH, USA) for surface area analysis. As screenshots do not retain inherent scale information, manual calibration was performed on each image using a known anatomical reference (e.g., the inciso‐apical length of the lateral incisor, as shown in Figure 1) to convert pixel dimensions into metric units (mm).

Graft area was measured using ImageJ (NIH, USA) based on standardized screenshots of digital impressions. As free gingival grafts often present with irregular, asymmetrical margins rather than rectangular shapes, surface area was determined by manually tracing the graft borders using the Freehand Selection Tool and Polygon Selection Tool, as demonstrated in Figure X.

To assess intra‐examiner repeatability, the same examiner performed graft area measurements on 10 sample images, twice, with a 2‐day interval between sessions. The intraclass correlation coefficient (ICC) was calculated as 0.92, indicating excellent intra‐examiner reliability.

#### 2.10.3 Clinical Data

**FIGURE 2:**
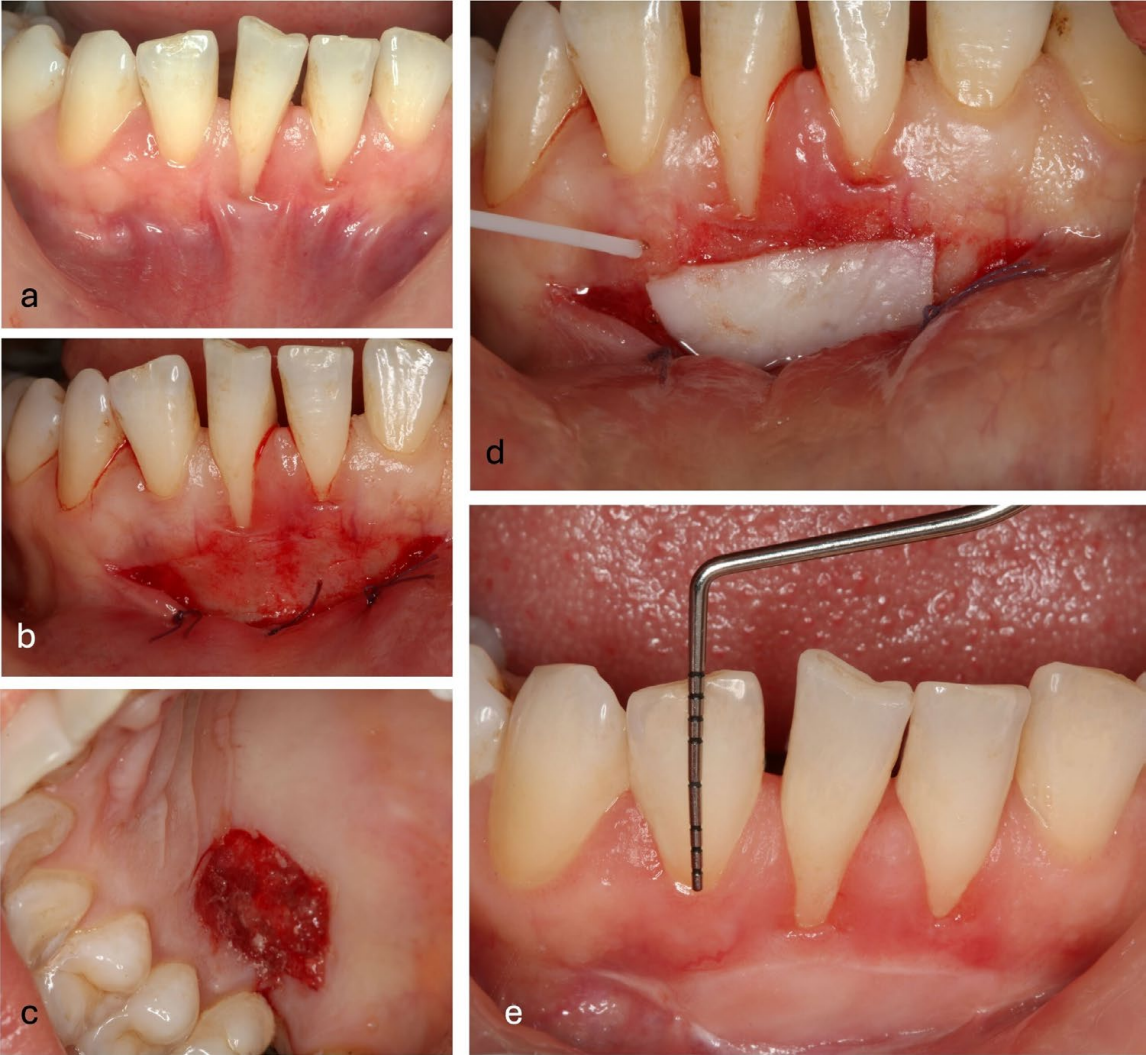
The intraoral photographs documenting the surgical procedure of the case illustrated in the digital surface area analysis in Figure 1 are presented in Figure 2.

Baseline assessments included age, gender, PI, PPD, and GI. Palatal thickness was measured preoperatively using an anesthesia needle with a silicone disk stop at the mesial, distal, and central graft regions, with an average value calculated. Gingival graft thickness was assessed immediately postoperatively and on day 30 at the distal, mesial, and central sites. The clinical procedure steps recorded through intraoral photographs for the case presented in the digital measurement of Figure 1 are shown in Figure 2.

Surgical duration was recorded for recipient bed preparation (from the initial incision to periosteal suturing), FGG harvesting (from the incision to hemostasis, with or without cyanoacrylate), and FGG stabilization (time to secure the graft).

Postoperative pain was assessed using VAS diaries, in which patients rated pain intensity on a 0–100 scale for 7 days, with 0 indicating no pain and 100 representing the worst imaginable discomfort.

### 2.11 Statistical Analysis

Statistical analysis was performed using SPSS v.23. Data normality was assessed with the Kolmogorov–Smirnov test. Independent t‐tests were used for normally distributed variables, while the Mann–Whitney U test was applied to nonparametric data. Repeated‐measures ANOVA was conducted for time‐dependent comparisons, and categorical variables were analyzed using the χ^2^ test. Results were reported as mean ± standard deviation (SD) for parametric data, median (min–max) for nonparametric data, and frequency (percentage) for categorical data. A *p*‐value < 0.05 was considered statistically significant.

## 3 Results

### 3.1 Participants and Time of Surgery

The test group consisted of 9 males and 11 females (mean age: 31.9 ± 3.80 years), while the control group included 10 males and 10 females (mean age: 32.8 ± 4.58 years). No significant differences were observed between the groups in PI, GI, PPD, or palatal thickness. Recipient bed preparation time was comparable between the two groups. However, FGG stabilization and total operation time were significantly shorter in the test group (*p* < 0.001 and *p* = 0.001, respectively), whereas donor site preparation took longer (*p* < 0.001) (Table 1). Additionally, willingness to repeat the treatment was significantly higher in the test group (*p* = 0.026) (Table 1).

**TABLE 1:** Demographic characteristics and operative details, including periodontal parameters, procedure duration, and palatal thickness.

Parameters	Test mean ± SD	Control mean ± SD	*p* *
Number of patients	20	20	
Age (years)	31.9 ± 3.80	32.8 ± 4.58	0.503
PI	0.40 ± 0.19	0.39 ± 0.17	0.839
GI	0.23 ± 0.14	0.21 ± 0.13	0.557
PPD	1.46 ± 0.10	1.42 ± 0.11	0.232
Palatal Thickness (mm)	2.71 ± 0.60	2.72 ± 0.91	0.967
Distribution of total operation time by stages (min)			
Preparation of the recipient bed	5.45 ± 0.95	5.7 ± 0.73	0.356
FGG harvesting–donor site preparation	16.1 ± 0.97	14.9 ± 1.07	**0.001**
FGG stabilization	6.3 ± 0.73	18.7 ± 1.66	**< 0.001**
Total duration of the operation	27.85 ± 2.01	39.3 ± 2.25	**< 0.001**

* Unpaired *t* test, the bold characters indicate statistically significant values (*p* < 0.05).

### 3.2 Laser Doppler Flowmetry Measurements

LDF values at both donor and recipient sites changed significantly over time in both groups (*p* < 0.001). At the donor site, values were highest on day 10 and lowest at baseline. Similarly, recipient site values peaked on day 10, with the lowest measurements recorded at baseline and day 30.

**FIGURE 3:**
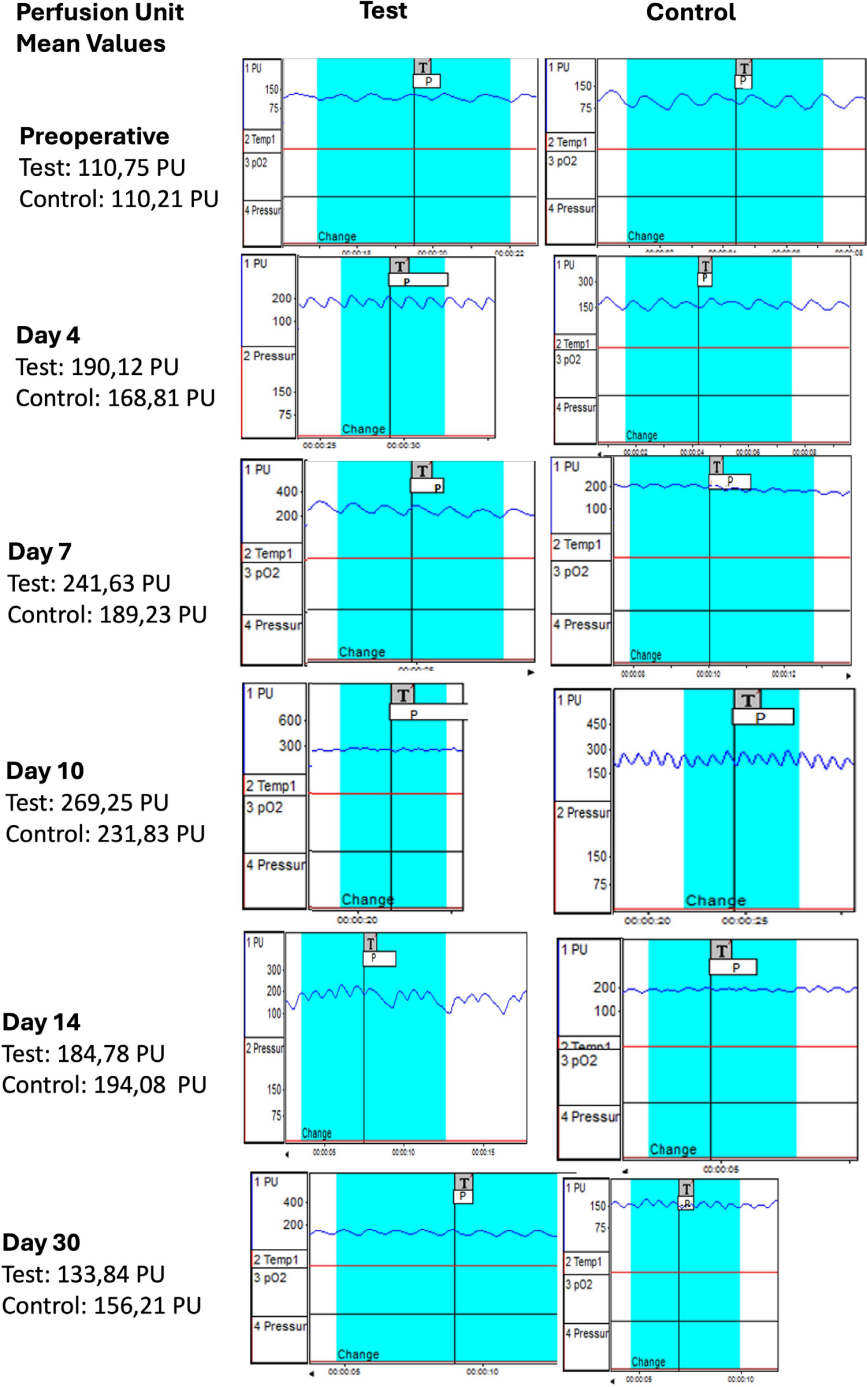
Comparative LDF screen captures from the donor sites of one representative patient from the test and control groups are presented.

At the donor site, the test group exhibited significantly higher LDF values than the control group on days 4 and 7 (*p* = 0.002, *p* < 0.001). However, after day 10, values were significantly higher in the control group (*p* = 0.002, *p* = 0.448, *p* = 0.005). Figure 3 presents comparative Laser Doppler Flowmetry (LDF) screenshots from the donor sites of one representative patient in each group (test and control), highlighting perfusion trends at multiple time points.

At the recipient site, LDF values were significantly higher in the test group on days 4 and 7 (*p* = 0.001, *p* = 0.042), but no significant differences were observed on days 10 and 30 (*p* > 0.05). On day 14, the control group exhibited significantly higher values (*p* < 0.001) (Table 2).

**TABLE 2:** Comparative Laser Doppler Flowmetry Measurements of Donor and Recipient Sites in Test and Control Groups During the Study Period.

	Test (*n* = 20)	Control (*n* = 20)	p**
LDF_donor site_			
preoperative	103.74 ± 17.68^e^	102.03 ± 20.41^e^	0.778
Day 4	192.99 ± 25.15^b^	165.29 ± 27.37^d^	**0.002**
Day 7	238.8 ± 30.03^d^	196.2 ± 23.65^b^	**< 0.001**
Day 10	272.43 ± 36.71^c^	236.76 ± 29.13^c^	**0.002**
Day 14	189.67 ± 14.22^b^	193.85 ± 19.76^b^	0.448
Day 30	130.14 ± 19.05^a^	149.11 ± 21.29^a^	**0.005**
p value*	< 0.001	< 0.001	
LDF_recipient site_			
preoperative	33.00 ± 7.63^a^	29.78 ± 10.12^a^	0.263
Day 4	165.74 ± 19.31^e^	143.89 ± 18.37^b^	**0.001**
Day 7	192.39 ± 25.91^d^	177.09 ± 19.71^d^	**0.042**
Day 10	206.70 ± 17.40^c^	209.62 ± 18.91^c^	0.615
Day 14	108.25 ± 17.18^b^	136.85 ± 15.82^b^	**< 0.001**
Day 30	33.45 ± 9.67^a^	35.37 ± 15.15^a^	0.636
p value*	< 0.001	< 0.001	

*Note*: ^a‐e^There is no difference between the times with the same letter in each group. *Repeat‐measures one‐way analysis of variance **Independent sample t test, the italic and bold written is *p* < 0.05 statistically significant.

### 3.3 FGG Digital Area and Thickness

**FIGURE 4:**
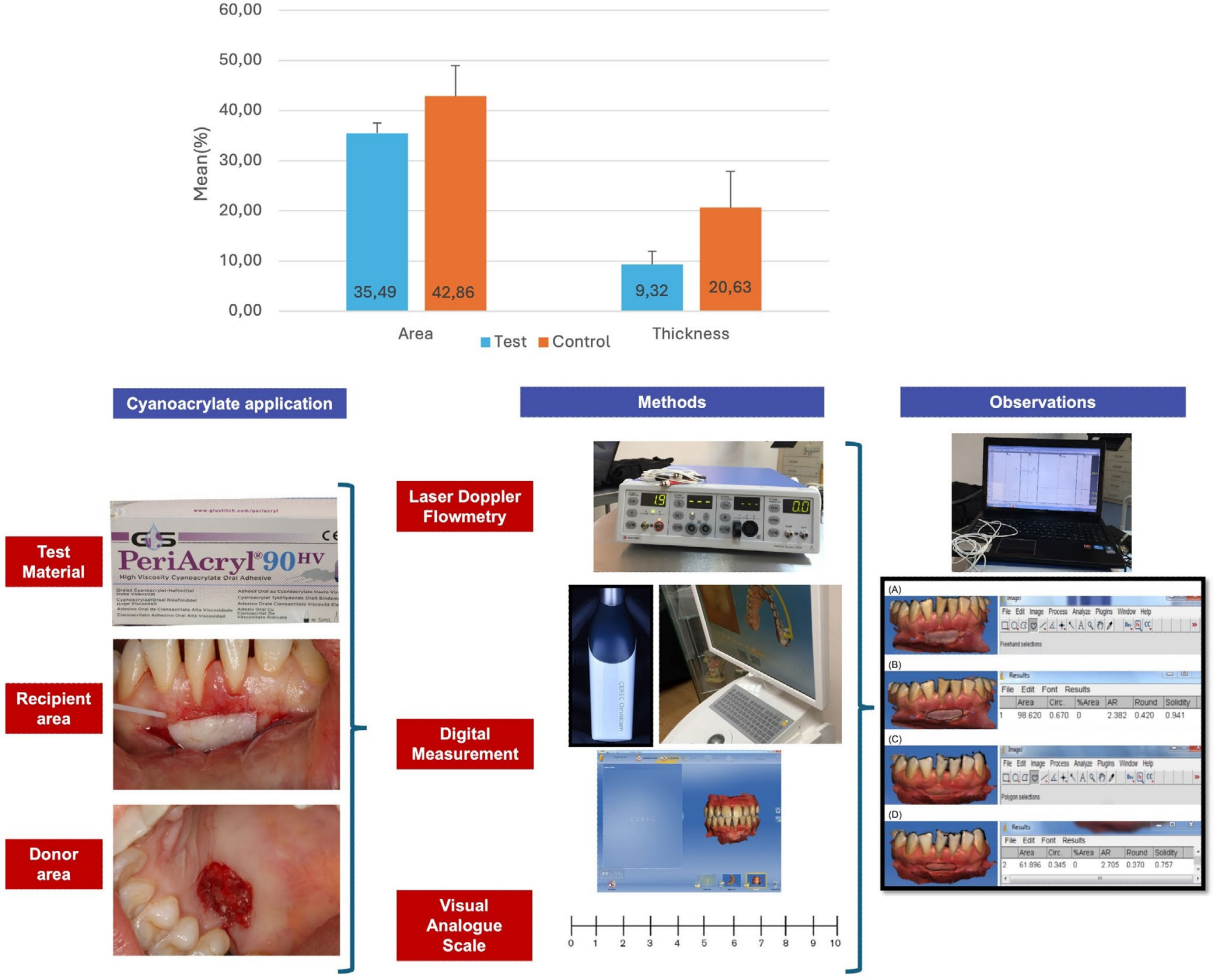
Changes in Area and Thickness of Free Gingival Graft: Comparison Between Test and Control Groups.

A significant difference was observed between the groups in FGG digital area change (35.49 vs. 42.86) and thickness change (9.32 vs. 20.63), both of which were lower in the test group (*p* < 0.001). Although baseline values were similar, day 30 measurements were higher in the test group (Table 3). Changes in the thickness of the free gingival graft, comparing the test and control groups, are illustrated in Figure 4.

**TABLE 3:** Evaluation of Free Gingival Graft Shrinkage: Area and Thickness Comparisons Between Test and Control Groups.

	Test	Control	p**
FGG area (mm^2^)			
Baseline	95.32 ± 2.5	94.92 ± 2.79	0.630
1st month	61.49 ± 2.26	54.19 ± 1.66	**< 0.001**
*p* value*	< 0,001	< 0,001	
Difference	35.49 ± 2.01	42.86 ± 2.62	**< 0.001**
**%**	37.23 ± 1.23	45.15 ± 1.79	**< 0.001**
FGG thickness (mm)			
Baseline	1.19 ± 0.16	1.2 ± 0.22	0.880
1st month	1.08 ± 0.16	0.94 ± 0.15	**0.010**
*p* value*	< 0,001	< 0,001	
Difference	9.32 ± 6.09	20.63 ± 7.21	**< 0.001**

*Note*: *Repeat‐measures one‐way analysis of variance **Independent sample t test.

### 3.4 Visual Analog Scores

VAS scores decreased at both recipient and donor sites from day 1 to day 7 in both groups. The test group exhibited significantly lower VAS values than the control group during the first 6 days (*p* < 0.05) (Table 4).

**TABLE 4:** Postoperative Pain Assessment Using Visual Analog Scale (VAS) at Donor and Recipient Sites in Test and Control Groups (Days 1–7).

	Day 1	Day 2	Day 3	Day 4	Day 5	Day 6	Day 7
Test (*n* = 20)							
Mean ± SD	2.4 ± 1.05	1.6 ± 0.82	1.15 ± 0.81	0.6 ± 0.50	0.4 ± 0.50	0.2 ± 0.41	0.05 ± 0.22
Median	3	1	1	1	0	0	0
Min‐max	1–4	1–4	0–3	0–1	0–1	0–1	0–1
Control (*n* = 20)							
Mean ± SD	3.75 ± 0.91	3.1 ± 0.97	1.95 ± 0.1	2.1 ± 0.64	1.3 ± 0.66	0.7 ± 0.47	0.35 ± 0.49
Median	4	3	2	2	1	1	0
Min‐max	2–5	2–5	1–4	1–3	0–2	0–1	0–1
*p value* *	**< 0.001**	**< 0.001**	**0.015**	**< 0.001**	**< 0.001**	**0.002**	0.108

* Mann Whitney U test, the bold written is *p* < 0.05 statistically significant.

## 4 Discussion

Graft vascularization continuity is critical in preventing shrinkage. Graft thickness, atraumatic technique, and rapid stabilization minimize contraction by protecting vessels from damage and dehydration. As each suture induces localized hematoma formation, reducing the number of sutures is recommended. Cyanoacrylate‐based adhesives provide low toxicity, cost‐effectiveness, ease of use, and high efficiency. They are used in periodontal surgery, including FGGs, to reduce suture‐related complications [17, 24]. Adhesive sealants serve as an alternative to sutures in mucogingival surgery by shortening surgical time, preserving healing, reducing discomfort and bleeding, and eliminating suture removal [19, 22]. Studies on cyanoacrylate (CA) based adhesives in palatal applications after free connective tissue grafts and closed surgical sites lack data on their impact on perfusion at both donor and recipient sites in FGG procedures [19, 22, 43]. This randomized controlled trial assessed CA's effect on graft contraction using digital impressions and its impact on perfusion and vascularization at recipient and donor sites. The study hypothesizes that CA facilitates early microvascular network formation, accelerating healing, reducing contraction, and enhancing vascularization at the donor site. To our knowledge, this is the first study comparing CA vs. sutures in FGGs using both LDF and digital impression techniques. This study differs from previous FGG research in three key aspects: the use of LDF, an objective and noninvasive method providing numerical data independent of patient perception; digital impression for graft contraction assessment; and the inclusion of gingival thickness as a selection criterion. Accurate interpretation of LDF data requires ensuring that perfusion values originate solely from the target tissue. If gingival thickness is < 0.75 mm, LDF may detect signals from the root and periosteum, potentially affecting perfusion measurements. To eliminate this confounding effect, a minimum of 1 mm gingival thickness was required. In preliminary cases treated before the study design was established, excessive tissue growth was observed when cyanoacrylate was applied to the apical graft corners. Consequently, these patients were not included in the study. To our knowledge, no previous study has reported cyanoacrylate‐induced tissue overgrowth in the literature. This study is the first to document this phenomenon, and histopathological evaluation confirmed that the observed tissue overgrowth was of inflammatory origin. As a precautionary measure, the cyanoacrylate application technique was revised accordingly. Cyanoacrylate was applied differently at the two surgical sites: on the donor site, it was used to fully cover the wound surface in the form of fine microdroplets, aiming to promote hemostasis and protect the wound; on the recipient site, it was limited to the coronal mesial and distal graft corners, forming a 2 mm‐wide adhesive layer to stabilize the graft while preventing apical tissue response. FGG stabilization with CA was three times faster than suturing, aligning with prior studies [43]. Our previous study demonstrated that CA enables immediate FGG stabilization without fully depleting its remaining blood perfusion, a concept first introduced in our research [3]. The linear healing parameter, introduced by Hopkins and Jamiesen, tracks two‐dimensional wound healing [44]. FGG shrinkage is defined as the graft margin's movement toward the wound center. Previous studies assumed shrinkage follows a linear contraction pattern, calculated via the rectangular area formula [26]. However, healing often causes uneven edge contraction, altering graft shape. Since shrinkage is nonlinear and does not follow a fixed axis, calculating contraction based on surface area rather than a linear model provides more accurate results.

The use of digital impression techniques for intraoral imaging to assess FGG shrinkage offers significant advantages in periodontal research. Unlike photographic imaging, digital impressions eliminate artifacts caused by patient movement and reduce variations between repeated measurements due to external factors over time. In this study, digital impression images allowed investigators to compare FGG shrinkage across different regions and determine the absolute area of graft contraction. Digital impression measurement eliminates movement artifacts and ensures standardized imaging. This is the first study assessing FGG shrinkage after CA application using digital impression, providing a scientific foundation for future research. Intraoral photographic assessments are subject to several limitations that can compromise measurement accuracy. Minor deviations in camera angulation or patient positioning can significantly affect the perceived dimensions of the graft. Furthermore, variations in lighting conditions—such as intensity, direction, and color temperature—between sessions may obscure graft margins and hinder consistent evaluation. To achieve accurate measurements, the use of a calibrated reference object (e.g., periodontal probe) is required within the field of view. In response to these limitations, digital impressions have been used as an alternative, offering standardized, reproducible, and user‐independent image acquisition.

Various studies on dermal analog contraction have utilized different macroscopic assessment methods. Truong et al. [45] used Image Tool 2.0 for planar surface area analysis, while Schneider et al. [46] and Böttcher‐Haberzeth et al. [47] analyzed Matriderm and Integra using ImageJ. Similarly, Philandrianos et al. [48] employed AutoCAD to compare acellular dermal analogs. ImageJ was used to measure macroscopic contraction and compare grafted areas within and between groups. Its application did not hinder comparisons with previous studies or affect the objective evaluation of findings. Since earlier research analyzed changes within each group, differences in assessment methods did not influence result interpretation.

Previous studies have reported that the vertical shrinkage of thin (FGGs at day 30 ranges between 45% and 47%) [24]. In this study, shrinkage in the CA group (37.23%) was significantly lower than in the control group (45.15%). These findings suggest that CA application is less traumatic than suturing, and rapid stabilization may help preserve remaining blood perfusion during fixation.

Regarding surface area reduction, the control group exhibited a contraction of approximately one‐third of its initial dimension, whereas in the CA group, this reduction was limited to one‐fourth Consistent with these findings, previous research on FGG shrinkage has also demonstrated that CA application results in significantly less contraction compared to conventional suturing techniques [25].

A review of studies assessing graft shrinkage reveals considerable variations in reported values. These discrepancies may be attributed to differences in measurement sensitivity, the absence of software‐assisted analysis, and the lack of digital impression methods that minimize variability in absolute area over time.

Barbosa et al. reported no significant shrinkage difference between CA (39.1%) and sutures (41.0%) [24]. This outcome may be attributed to variations in measurement precision, differences in study design, or an insufficient follow‐up duration. However, another study reported findings consistent with the present study, demonstrating reduced shrinkage in the CA group [25]. Tissue adhesives minimize contraction by enabling atraumatic, rapid fixation, supporting the avascular plasmatic circulation process. The time points selected for follow‐up in this study were based on histologic and clinical evidence of biologically significant phases in graft healing. Around day 4, vascular proliferation begins at the graft margins [49], while neovascularization peaks between days 5 and 10 and stabilizes within 2 to 4 weeks. Day 7 corresponds with early epithelial ridge formation and vascular bridging between recipient site and graft [49], while day 14 marks the development of mature epithelium with abundance of capillaries [50]. Oliver et al. reported that keratinization becomes clinically observable around day 28 [51]. Additionally, previous studies [26, 52] have shown that 50%–80% of total graft shrinkage occurs within the first 4 weeks. Therefore, selecting time points within this early healing window was considered optimal to capture the most dynamic changes in perfusion and surface area, making this period particularly suitable for LDF‐based monitoring and digital impression‐based analysis.

In most patients, cyanoacrylate applied to the edges of the graft at the recipient site remained intact until day 5, as it was exposed to less trauma from daily activities such as eating, drinking, and speaking compared to CA applied to the donor site. Moreover, CA may help protect the recipient site from mechanical trauma caused by lip movements and food intake, thereby enhancing patient comfort. Consistent with our findings, Galil et al. reported that tissue adhesives were resorbed by day 5, with their remnants undergoing phagocytosis, which in turn reduced the formation of multinucleated giant cells [53]. The higher LDF values observed at the donor site in the CA group on days 4 and 7, compared with the control group, suggest that CA exerts a protective role through its microbial barrier effect and facilitates early revascularization, thereby accelerating the healing process. Conversely, the higher LDF values in the control group on days 14 and 30 indicate that CA remained effective during the early healing phase and subsequently underwent controlled resorption. The lower perfusion values observed in the control group during the first 10 days further suggest a delay in healing compared with the CA group. Davis et al. found that CA promotes early blood accumulation, reducing recirculation time and accelerating healing [54]. Our findings support this concept, showing CA enhances early vascularization at the recipient site. Consistent with the findings of Vastani et al., CA adhesives have been shown to support early wound healing and, compared with standard suture closure, cause less intraoperative and postoperative discomfort when applied to intraoral wounds [55]. During the healing process of FGGs, perfusion peaked on day 10, at which point it became comparable between groups. Although higher perfusion values were observed in the control group on day 14, these values subsequently declined and equalized by day 30. The absence of a significant difference between groups on day 30 suggests that CA stabilization does not influence tissue perfusion once healing is complete. In contrast, suture‐induced perfusion changes appear to be limited to the first 10 days of healing. These findings indicate that CA stabilization may reduce the risk of necrosis in transplanted tissue and accelerate the healing process. Evaluation of VAS scores in the early postoperative period revealed that patients experienced pain and postoperative discomfort up to day 7; however, pain was significantly lower in the CA group until day 6. Stavropoulos et al. reported that CA did not result in a significant difference in patient comfort or postoperative pain. This discrepancy may be attributed to the secondary wound area formed after FGG harvesting in our study, which likely caused greater postoperative pain compared with the donor site in a connective tissue graft procedure performed using the single‐incision technique. Therefore, the statistically significant reduction in postoperative pain observed in the CA group in this study should be interpreted in this context. Additionally, patients in the CA group reported that early postoperative numbness and bleeding at the recipient site gradually decreased and had completely resolved by day 7. In our study, graft shrinkage and perfusion values were clinically compared in the early postoperative period. Due to the ease of measurement and lip retraction during probe stabilization with cyanoacrylate, the assessment was limited to the anterior region. The stabilization of free gingival grafts using tissue adhesives appears to support the ‘avascular plasmatic circulation’ process by providing atraumatic and rapid fixation, thereby potentially reducing graft shrinkage. However, further well‐designed randomized clinical trials with larger sample sizes are needed to evaluate perfusion values in the posterior gingival region and to further investigate the effects of cyanoacrylate.

Findings from this study suggest that n‐butyl cyanoacrylate, compared with sutures, may serve as a single‐use, reliable, fast, and easily applicable alternative for FGG procedures. Additionally, it may function as an effective wound dressing and stabilizer by enhancing perfusion in non‐infected, painless wound areas, reducing graft shrinkage, preserving the integrity of transplanted tissue, and accelerating wound healing.

This study provides novel evidence regarding the dual‐site effects of cyanoacrylate in free gingival graft procedures. By combining digital surface area analysis with LDF, it objectively demonstrates the cyanoacrylate ‘s impact not only on graft shrinkage and healing dynamics at the recipient site but also on microvascular perfusion at the donor site. Although previous literature has primarily relied on subjective clinical outcomes, this is the first study to present quantitative data from both donor and recipient sites. Through the use of LDF, this study not only assessed the extent of graft shrinkage but also quantitatively monitored the underlying microvascular processes contributing to this outcome. In particular, in areas where cyanoacrylate was applied, LDF enabled real‐time tracking of perfusion changes, inflammatory response, and tissue regeneration at the donor site. The objective data provided by LDF thus offered valuable insight into the biological safety and vascular compatibility of different graft stabilization methods.

## Author Contributions

Zeynep Turgut Çankaya contributed to study design, data collection, and manuscript writing. Sühan Gürbüz performed statistical analysis and critically revised the manuscript. Evşen Tamam performed the digital impression part.

## Conflicts of Interest

The authors declare no conflicts of interest.

## Data Availability

The data that support the findings of this study are available on request from the corresponding author. The data are not publicly available due to privacy or ethical restrictions.
